# Clinical outcomes of zirconia implants: a systematic review and meta-analysis

**DOI:** 10.1007/s00784-023-05401-8

**Published:** 2023-12-23

**Authors:** Parvin Mohseni, Ahmad Soufi, Bruno Ramos Chrcanovic

**Affiliations:** 1 Faculty of Odontology, Malmö University, Malmö, Sweden; 2 Department of Oral and Maxillofacial Surgery and Oral Medicine, Faculty of Odontology, Malmö University, Carl Gustafs Väg 34, 214 21 Malmö, Sweden

**Keywords:** Dental implants, Zirconia implants, Ceramic implants, Zirconium oxide, Survival, Marginal bone loss, Systematic review, Meta-analysis

## Abstract

**Purpose:**

To assess the clinical outcomes of zirconia dental implants based on an updated systematic literature review.

**Methods:**

An electronic search was performed in three databases, last updated in June 2023, supplemented by hand searching. The eligibility criteria were clinical studies reporting patients rehabilitated with zirconia implants. The cumulative survival rate (CSR) of implants was calculated. A meta-analysis for marginal bone loss (MBL) under different follow-up times and a meta-regression assessing the relationship between mean MBL and follow-up were done.

**Results:**

Twenty-five studies were included (4017 implants, 2083 patients). Seven studies had follow-up longer than 60 months. 172 implants failed, after a mean of 12.0 ± 16.1 months (min–max 0.3–86.0), of which 47 early failures, and 26 due to implant fracture, the majority in narrow-diameter implants. The 10-year CSR was 95.1%. Implants with coronal part prepared by drills presented statistically significant lower survival than non-prepared implants (*p* < 0.001). Two-piece implants presented lower survival than one-piece implants (*p* = 0.017). Implants discontinued from the market presented lower survival than the commercially available ones (*p* < 0.001). The difference in survival was not significant between implants in maxilla and mandible (*p* = 0.637). The mean MBL fluctuated between 0.632 and 2.060 mm over long periods of observation (up until 132 months). There was an estimated MBL increase of 0.005 mm per additional month of follow-up.

**Conclusion:**

Zirconia implants present high 10-year CSR and short-term low MBL.

The review was registered in PROSPERO (CRD42022342055).

**Clinical relevance:**

The clinical outcomes observed for zirconia dental implants are very promising, although these have not yet been extensively studied as titanium alloy implants.

**Supplementary Information:**

The online version contains supplementary material available at 10.1007/s00784-023-05401-8.

## Introduction

As of today, titanium and titanium alloys are the materials most often utilized in implant manufacture, and they have greatly improved clinical outcomes regarding tooth replacement. Because of their biocompatibility, advantageous mechanical characteristics, and well-established positive outcomes, these materials have achieved wide applicability [1, 2]. Many investigations have shown the long-term efficacy of dental implants made of titanium [3, 4].

The major disadvantage of titanium is its dark gray hue, which is frequently visible through the peri-implant mucosa and compromises esthetic results when a thin mucosal biotype is present [5, 6]. Thus, compromise in esthetics may result from poor soft tissue health or gingival recession. This may become more critical when the maxillary incisors are affected [7, 8].

Possible unfavorable responses to the metal titanium are another issue. Numerous investigations came to the conclusion that titanium exposure could cause hypersensitivity, although conclusive proof is still lacking [9–13].

These disadvantages led to the adoption and development of other materials to be used in dentistry, such as ceramics [8]. Ceramics have steadily gained popularity in the dental sector due to esthetic demands. They are now utilized to manufacture not only dental prostheses but also dental implants. The choice for materials such as yttria-tetragonal zirconia polycrystal (Y-TZP) ceramics was influenced by some in vitro studies, one of which observed that zirconia implants presented mean fracture strength values within limits of clinical acceptance [14], although later on it was recommended that zirconia implants should be toughened with alumina instead (alumina-toughened zirconia [ATZ], with 20 wt% alumina), due to its increased mechanical stability compared to Y-TZP [15]. Moreover, according to the results of a biomechanical animal study, acid-etched zirconia implants have the potential to enhance bone apposition resulting in removal torque testing with similar values to sandblasted and acid-etched titanium implants [16]. The results of a histomorphometric animal study showed that there was no difference in osseointegration between acid-etched zirconia and sandblasted and acid-etched implants of titanium regarding peri-implant bone density and bone-implant contact ratio [17].

Clinical studies of zirconia dental implants have been recently more frequently published in the literature. However, concerns regarding their long-term survival and occurrence of fractures are still present. As of 2017, many questions on the use of zirconia for dental implants were still unanswered [18, 19]. A review on the subject was recently published [20], although with very restricted inclusion criteria, which limited the number of included studies. Moreover, survival analysis of zirconia implants in relation to preparation or not of their coronal part with drills, as well as in relation to implants discontinued from the market or still commercially available, was not performed. Furthermore, the estimated mean marginal bone loss (MBL) over different periods of follow-up was not calculated. It was therefore the purpose of the present review to assess the clinical outcomes of zirconia implants based on an updated and more comprehensive systematic review of the literature.

## Materials and methods

This study followed the PRISMA Statement guidelines, also valid for the abstract [21]. Registration in PROSPERO was undertaken with the registration number CRD 42022342055.

The focused question was later slightly modified in comparison to the PROSPERO registry, in order to more precisely accommodate the outcomes investigated in the present review.

### Focused question

The focused question was: What is the survival rate, the prevalence of implant fracture, and MBL around zirconia implants in patients being rehabilitated with implant-supported prostheses?

### Search strategies

An electronic search without time restrictions was undertaken in October 2021, with a complementary update search in June 2023, in the following databases: PubMed/Medline, Web of Science (in “all databases”), and Science Direct. The following terms were used in the search strategies:

(“dental implant” OR “oral implant”) AND (“zirconia implant” OR “zirconium implant” OR “ceramic implant” OR “zirconia oral implant” OR “zirconia-based ceramic dental implant”)

A manual search was performed in the following journals: *Clinical Implant Dentistry and Related Research, Clinical Oral Implants Research, European Journal of Oral Implantology, Implant Dentistry, International Journal of Implant Dentistry, International Journal of Oral and Maxillofacial Implants, International Journal of Oral Implantology, International Journal of Prosthodontics, Journal of Clinical Periodontology, Journal of Oral Implantology, Journal of Periodontology, Journal of Prosthetic Dentistry, Journal of Prosthodontics,* and *Journal of Prosthodontic Research*. The reference list of the identified studies and the relevant reviews on the subject were also checked for possible additional studies. Grey literature was not searched.

### Inclusion and exclusion criteria

Eligibility criteria included clinical studies, either randomized or not, providing information on implant failure rates in any group of patients receiving zirconia dental implants. Studies that investigated zirconia implants that were manufactured to copy the root anatomy after laser scanning of the extracted tooth were excluded. Exclusion criteria also comprised case reports, technical reports, animal studies, in vitro studies, and reviews papers.

### Study selection

The titles and abstracts of all reports identified through the electronic searches were screened independently by two reviewers (PM, AS). For studies appearing to meet the inclusion criteria, or for which there were insufficient data in the title and abstract to make a clear decision, the full report was obtained. The full text assessment was carried out independently by two reviewers. Any disagreements were solved by discussion and if needed by a third reviewer (BRC).

RefWorks Reference Management Software (Ex Libris, Jerusalem, Israel) was used in order to detect duplicate references in different electronic databases.

### Risk of bias within studies

Risk of bias within studies was carried out according to the Quality Assessment Tool for Case Series Studies of the National Institutes of Health [22]. The NIH tool calculates the study quality on the basis of nine criteria. The ratings on the different items were used by the reviewers to assess the risk of bias in the study due to flaws in study design or implementation. The studies were classified as “good,” “fair,” or “poor” quality. In general terms, a “good” study has the least risk of bias, and results are considered to be valid. A study rated as “fair” is susceptible to some bias, but deemed not sufficient to invalidate its results. The fair quality category is likely to be broad, so studies with this rating will vary in their strengths and weaknesses. A “poor” rating indicates significant risk of bias. Studies of “good” quality were judged to have at least 7 points.

### Definitions

Zirconia dental implants were defined as those being composed of zirconium dioxide (ZrO_2_) [23].

An implant was considered a failure if presenting signs and symptoms that led to implant removal, i.e., a lost implant. Implant failure could be either early (the inadequacy of the host to establish or promote osseointegration in the early stages of healing) or late (the failure of either the established osseointegration or function of dental implants) [24, 25]. Fracture of an implant was also considered a failure [26].

Marginal bone loss was defined as loss, in an apical direction, of alveolar bone marginally adjacent to the dental implant, in relation to the marginal bone level initially detected after the implant was surgically placed [27]. Only studies using the long-cone parallel technique for periapical radiographs were considered.

### Data extraction

From the studies included in the final analysis, the following data were extracted (when available): year of publication, study design and setting, number of patients, patients’ age, implant healing period, implants used (model and brand), jaws receiving implants (maxilla and/or mandible), jaw region (anterior/posterior), number of failed and placed implants, occurrence of implant fracture, type of prosthetic rehabilitation, and follow-up time. When needed, authors were contacted for additional information when data were missing.

### Analyses

The mean, standard deviation (SD), and percentage were calculated for several variables, from individual participant data that were collected and entered into the statistical software file. The log-rank (Mantel-Cox) test was used to compare the survival distributions of implants between one- and two-piece implants, between implants placed in the maxilla and in the mandible, between implants that had their coronal part prepared and not prepared by a drill, between implants commercially available and discontinued from the market, as well as for the occurrence of implant fracture between implants with their abutment part prepared or not with diamond drills. The interval survival rate (ISR) of implants was calculated using the information for the period of failure extracted from the included studies, and the cumulative survival rate (CSR) was calculated over the maximal period of follow-up reported, in a life-table survival analysis. The degree of statistical significance was considered *p* < 0.05. These data were statistically analyzed using the SPSS version 28 software (SPSS Inc., Chicago, IL, USA).

A meta-analysis applying the DerSimonian-Laird random-effects method [28] was performed to calculate the estimated MBL under different follow-up times. The *I*^2^ statistic was used to express the percentage of the total variation across studies due to heterogeneity, with 25% corresponding to low heterogeneity, 50% to moderate and 75% to high. A meta-regression assessing the relationship between mean MBL and follow-up was performed. The data were analyzed using the statistical software OpenMeta[Analyst] [29].

## Results

### Literature search

**Fig. 1:**
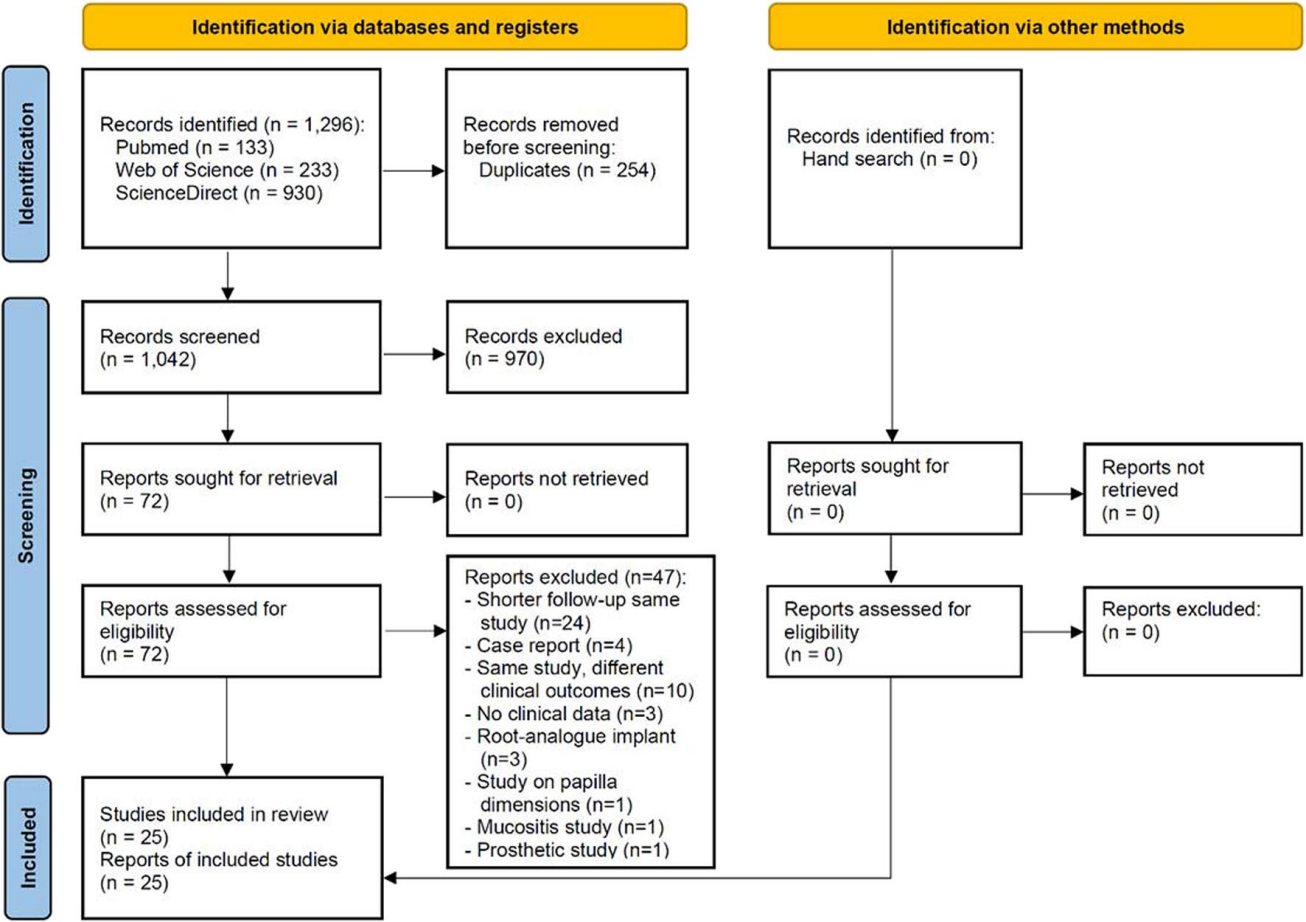
Study screening process

The study selection process is summarized in Fig. 1. The search strategy resulted in 1296 papers (133 in Pubmed/Medline, 233 in Web of Science, and 930 in Science Direct). A number of 254 articles were cited in more than one research of terms (duplicates). Of the resulted 1042 studies, 970 were excluded for not being related to the topic. Hand-searching of selected journals did not yield additional papers. The full-text reports of the remaining 72 articles led to the exclusion of 47 because they did not meet the inclusion criteria: shorter follow-up report with an already published longer follow-up report with the same cohort group of patients (*n* = 24), case report (*n* = 4), same study but reporting on different clinical outcomes (*n* = 10), publications with not enough available clinical data (*n* = 3), use of zirconia root-analog implants (*n* = 3), study on papilla dimensions (*n* = 1), study on mucositis (*n* = 1), and study on prosthetic maintenance (*n* = 1). Thus, a total of 25 publications were included in the review [30–54] (see list of references in Supplementary Material).

### Description of the studies

Table 1 presents the summarized data of the included studies and Table S1 (see Supplementary Material) present the detailed data of each included study. The 25 publications reported 4017 zirconia implants in 2083 patients. The patients consisted of 851 (42.4%) men and 1,157 (57.6%) women, with no available information on sex for 75 patients. The patients received a mean of 1.91 ± 1.51 (range, 1–14) implants. Seven out of the 25 studies had a follow-up longer than 60 months.

**Table 1:** Summarized data of the included studies—patients rehabilitated with implant-supported prosthesis on zirconia implants

Variable	
Patients (*n*)/implants (*n*)	2083/4017
Age (years), mean ± SD (min–max)	51.2 ± 14.7 (18**–**90)
Sex, *n* (%)	
Men/women	851 (42.4) /1,157 (57.6)
Not available	75
Implants per patient (*n*), mean ± SD (min–max)	1.91 ± 1.51 (1**–**14)
Implant type, *n* (%)	
One-piece	3818 (95.0)
Two-piece	199 (5.0)
Implant location, *n* (%)	
Jaw	
Maxilla	2264 (58.1)
Mandible	1,632 (41.9)
Not available	121
Region	
Anterior	850 (24.8)
Posterior	2572 (75.2)
Not available	595
Healing time (months), mean ± SD (min–max)	2.66 ± 1.79 (0**–**11)
Immediate loading, number implants (%)	980 (24.4)
0.5–3 months, number implants (%)	2097 (52.2)
4–6 months, number implants (%)	911 (22.7)
> 6 months, number implants (%)	29 (0.7)
Prosthesis type, *n* (%)	
Single crown	1326 (85.0)
FDP (2–5 units)	150 (9.6)
Overdenture	84 (5.4)
Not available	2457
Follow-up (months), mean ± SD (min–max)	
Implants	68.8 ± 52.5 (0.3**–**180.0)
Prostheses	66.8 ± 52.4 (1.0**–**177.0)
Preparation of the implant coronal part, *n* (%)	
No	3253 (81.0)
Yes	764 (19.0)
Implant failure (*n*), failure/total (%)	
Implant level	172/4017 (4.3)
Patient level	141/2083 (6.8)
One-piece implant	157/3,818 (4.1)
Two-piece implant	15/199 (7.5)
Commercially available	78/3,182 (2.5)
Not commercially available^a^	94/835 (11.3)
Preparation of the implant coronal part	
No	115/3,253 (3.5)
Yes	57/764 (7.5)
Time of failure (months), mean ± SD (min–max)	12.0 ± 16.1 (0.3**–**86.0)
Early failures^b^, number/total (%)	47/115 (40.9)
Implant fracture^c^, number/total (%)	26 ^d^/172 (15.1)
One-piece implant	25/3,818 (0.7)
Two-piece implant	1/199 (0.5)
Implant coronal part not prepared	8/3,245 (0.2)
Drill-prepared coronal part	18/746 (2.4)
Lost implant replaced by a new one^e^, number/total (%)	36/54 (66.7)
Implant location ^e^, *n* (%)	
Maxilla anterior	41 (26.8)
Maxilla posterior	45 (29.4)
Mandible anterior	26 (17.0)
Mandible posterior	41 (26.8)
Implant system, *n* (%)	
CeraRoot C^a^	249 (6.2)
CeraRoot ICE ^**f**^	2114 (52.6)
CeraRoot UC ^a^	249 (6.2)
Nobel Biocare ZiUnite^a^	122 (3.0)
Own manufactured^a^	121 (3.0)
Southern Implants^a^	77 (1.9)
Straumann PURE Ceramic^f^	374 (9.3)
VITA ceramic.implant^f^	71 (1.8)
VOLZIRKON1^a^	22 (0.6)
VOLZIRKON2^a^	22 (0.6)
White-SKY^f^	72 (1.8)
Z-Look3^a^	338 (8.4)
ZERAMEX T^a^	49 (1.2)
Ziraldent FR1^f^	53 (1.3)
Ziterion^a^	16 (0.4)
ZV3^a^	68 (1.7)

*FDP*, fixed dental prosthesis

^a^Implants that were discontinued by the manufacturers, namely, no longer produced. Or implants that were manufactured only for the study

^b^Failure up to the abutment connection; 57 out of the 172 failed implants were submitted to the immediate loading protocol

^c^Percentage from the implant cases that failed

^d^Eighteen out of the 26 implant fractures (69.2%) were observed in the study of Roehling et al. [51]

^e^For the cases with available information

^f^Implants commercially available

There were 172 implant failures, with the great majority of them occurring within the first year after implant installation (Table 2). Of these 172 failures, 26 were due to implant fracture [32, 34, 36, 42, 48, 50, 51], of which 25 fractures occurred in implants that are no longer commercially available: 18 Z-Look3, 3 Southern implants, 1 Nobel Biocare ZiUnite, 1 Zeramex T, 1 Volzirkon1, and in one case the fracture occurred in an implant which was manufactured only to be used in a study. The only report of a fractured implant still commercially available happened in a CeraRoot ICE. These 26 fractured implants represent 0.65% of the 4017 implants. Eighteen out of the 26 implant fractures (69.2%) were observed in the study of Roehling et al. [51]. Fifteen out of these 18 fractures in the study of Roehling et al. [51] occurred with implants of a diameter of 3.25 mm.

**Table 2:** Life-table survival analysis showing the cumulative survival rate of zirconia implants

Interval start time (years)	Number entering interval	Number withdrawing during interval	Number exposed to risk	Implant failures	ISR (%)	CSR (%)	SE
0	4017	31	4001.5	124	96.9	96.9	0.27
1	3862	799	3462.5	24	99.3	96.2	0.30
2	3039	248	2915.0	10	99.7	95.9	0.32
3	2781	665	2448.5	2	99.9	95.8	0.33
4	2114	113	2057.5	1	100.0	95.8	0.33
5	2000	462	1769.0	9	99.5	95.3	0.36
6	1529	67	1495.5	0	100.0	95.3	0.36
7	1462	206	1359.0	2	99.9	95.1	0.38
8	1254	210	1149.0	0	100.0	95.1	0.38
9	1044	138	975.0	0	100.0	95.1	0.38
10	906	151	830.5	0	100.0	95.1	0.38
11	755	152	679.0	0	100.0	95.1	0.38
12	603	105	550.5	0	100.0	95.1	0.38
13	498	160	418.0	0	100.0	95.1	0.38
14	338	226	225.0	0	100.0	95.1	0.38
15	112	112	56.0	0	100.0	95.1	0.38

*ISR*, interval survival rate (survival rate within each interval)

*CSR*, cumulative survival rate (cumulative proportion surviving at end of interval)

*SE*, standard error

There was available information about the location of the failed implants for 153 cases (89.0%) out of the 172 failures. The failure rate was higher among two-piece implants, and the difference in survival was statistically significant (*p* = 0.017; log-rank test). There was no statistically significant difference of implant survival between maxilla and mandible (*p* = 0.637; log-rank test).

There was no information in the publications if the implant failures occurred in women and/or men. Therefore, an analysis of correlation about failure and sex was not possible. The same was true for patient’s age.

Preparation of the one-piece implant abutment coronal part with a diamond drill was reported in 8 studies [33, 35, 38, 39, 44, 47, 51, 53], and the difference in survival as well as in the occurrence of implant fracture between implants with their coronal (abutment) part prepared or not prepared by a drill was statistically significant (*p* < 0.001 and *p* < 0.001, respectively; log-rank test), favoring non-prepared implants.

The difference in survival between implants that are still commercially available and implants that were discontinued (or that were manufactured only for the study) was statistically significant (*p* < 0.001; log-rank test), favoring commercially available implants.

Table 3 shows the results of the meta-analyses for the outcome MBL under different follow-ups. The forest plots of these analyses are presented in the Supplementary Material (Figures S1–S7).

**Table 3:** DerSimonian-Laird random-effects model analysis for MBL under different follow-ups

Follow-up (months)	Studies*/implants (*n*)	MBL estimate (95% CI) (mm)	SE	*p* value	Heterogeneity
2–6	9/333	0.948 (0.641, 1.256)	0.157	< 0.001	*τ*^2^ = 0.196, *p* < 0.001, *I*^2^ = 91.941
12–15	15/697	0.632 (0.434, 0.830)	0.101	< 0.001	*τ*^2^ = 0.130, *p* < 0.001, *I*^2^ = 93.299
18–24	5/183	1.021 (0.274, 1.769)	0.381	0.007	*τ*^2^ = 0.704, *p* < 0.001, *I*^2^ = 99.577
30–36	7/413	0.892 (0.429, 1.354)	0.236	< 0.001	*τ*^2^ = 0.354, *p* < 0.001, *I*^2^ = 96.225
48	2/36	2.060 (1.885, 2.235)	0.089	< 0.001	*τ*^2^ = 0.045, p = 0.048, *I*^2^ = 81.493
60	5/217	0.958 (0.705, 1.211)	0.129	< 0.001	*τ*^2^ = 0.068, *p* < 0.001, *I*^2^ = 89.234
70–132	6/321	1.175 (0.876, 1.473)	0.152	< 0.001	*τ*^2^ = 0.123, *p* < 0.001, *I*^2^ = 94.685

*MBL*, marginal bone loss; *95% CI*, 95% confidence interval; *SE*, standard error

*When data on MBL for all implants in a study was not available (as a global mean value), then data on the mean value of the different sub-groups was entered. In these cases, each sub-group was considered one “study”

**Fig. 2:**
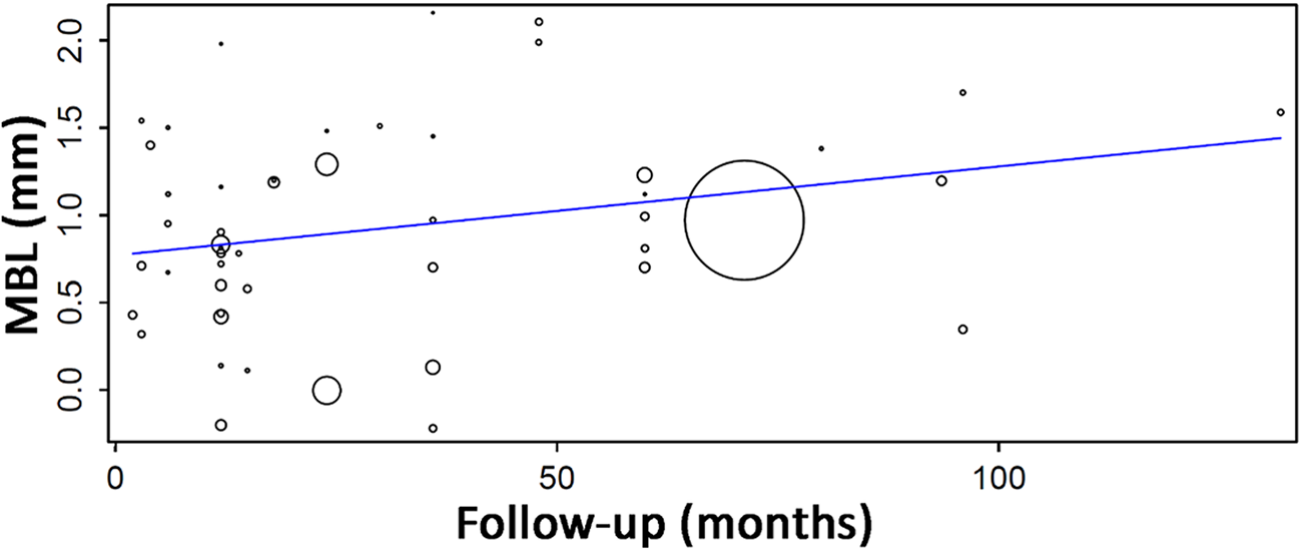
Scatter plot for the meta-regression with the association between follow-up (in months) and mean marginal bone loss (MBL). Positive values mean bone loss, while the negative values mean bone gain. Every circle represents a study or a different follow-up point in a same study, and the size of the circle represents the weight of the study in the analysis

A meta-regression considering the effect of follow-up on the mean MBL (Fig. 2) resulted in the following first-degree equation:

$$y=0.769+0.005x,$$

where:

Intercept: 0.769 (0.547, 0.990), standard error 0.113, *p* < 0.001
Follow-up: 0.005 (0.000, 0.010), standard error 0.003, *p* = 0.048

There was an estimated increase of 0.005 mm in MBL for every additional month of follow-up, with statistical significance.

### Risk of bias within studies

All included studies were classified as “good” (Table S2—see Supplementary Material). In most cases, the main issues in the publications were related to not well-described statistical methods and to the inclusion of non-consecutive patients in the studies.

## Discussion

According to the results of the present review, zirconia implants presented an estimated CSR of 95.1% after 10 years. The rate of implant fracture among the failed implants was 15.1% (26 fractured implants out of the 172 implant failures).

Zirconia implants seem to have a high estimated CSR after 10 years. However, these results of the life table analysis should be interpreted with caution, as most of the cases were followed up for a few years only. There was no change in the CSR from the year 8, as there were no recorded failures. Moreover, numbers entering the interval were low from year 10 and the censored numbers were proportionally high, reducing the confidence of the outcomes [55]. It is important to take note that the most recent observations are the least reliable because of the decreasing number of patients at risk for the event of interest [56], namely, implant failure.

The 26 fractured implants represent 0.65% of the total number of zirconia implants of the included studies, which is similar to the fracture rate of 0.44% observed in a study with more than 10,000 titanium implants [26]. The occurence of implant fractures can berelated to the material. An in vitro study [57] examining fracture strength has addressed a further issue with zirconia dental implants. It demonstrated that zirconia implants’ fracture strength resistance can be decreased by both pretreatment and cyclic loading, although even the lowest values of mean fracture strength of the implants used in the study seemed to withstand average occlusal forces [58]. However, there is a great variation in normal chewing forces, which for the posterior dentition may range from 110 to 125 N, and in the anterior dentition from 60 to 75 N [59, 60], variation that may be related to many factors, such as age, sex, muscle size, degree of edentulism, bone shape, and parafunction [61]. And it is not known whether these patients who presented implant fracture were bruxers, as nocturnal bite force during bruxism can exceed the amplitude of maximum voluntary bite force during the daytime [62]. Moreover, ceramic materials might undergo aging effect over the years in the oral environment [63]. The stability of the Y-TZP ceramic may be problematic under clinical conditions where the material is exposed for extended periods of time to thermal and cyclic mechanical stresses in a chemically active aqueous environment [64]. Here is important to point out that almost all fractures happened in implants that have been discontinued from the market by the manufacturers. This might possibly be related to differences in distinct generations of zirconia implants, with higher failure and fracture rates occurring in the early generation, being supposedly related to implant design [65] and/or the zirconia used [15].

The fractures could be associated with the diameter of the implant, as a considerable number of fractures occurred in implants of narrow diameter. The results of a review on in vitro investigation of fracture resistance of zirconia implants showed that zirconia implants of narrower diameter (3.0–3.3 mm) present much lower bending moments at the time point of fracture than implants or regular (3.8–4.4 mm) and wide diameter (4.5–5.0 mm) [66]. An in vitro study, published after the aforementioned review, evaluated and compared the fatigue resistance and fracture strength of zirconia implants of different diameters (3.0 and 3.7 mm), with straight or 15°-angled abutments. It was observed that all 3.0 mm diameter implants failed the fatigue test, with better performed demonstrated by the implants of wider diameter [67]. Specifically in relation to the implant that showed the highest prevalence of fracture, the Z-Look3 of diameter 3.25 mm, a failure analysis study had already recommended that this implant should no longer be used clinically, and that modification of the implant geometry was needed [65]. Moreover, a fractographic analysis study observed that the large grit alumina sandblasting of the Z-Look3 implants created deep v-notch type defects on the surface, which may act as starter cracks in stress concentration, directly related to fracture origin of the recovered clinically fractured implants investigated in the study [68].

Moreover, the coronal part of the implants was prepared by drills in some studies, and these presented a lower survival than non-prepared implants. Some one-piece zirconia implants might need to be prepared, as with natural teeth, in order to better fit prosthetic angulation requirements, as their coronal “abutment” part is fixed, not being possible choose abutments of different angulations to correct misalignment, as it is commonly seen in two-piece dental implants of titanium alloys. Values of fracture strength of zirconia may also vary if prepared, as grinding of zirconia deteriorates its physical properties by promoting a *t–m* transformation [69, 70]. A greater amount of monoclinic phase on the surface of the material after grinding can often lead to microcracking [71], which could extend deep into the subsurface, acting as internal stress concentrators causing the initiation of a crack throughout the material [57]. This was reflected in the clinical results, as most of the 26 fractured implants had their coronal abutment part prepared by drills.

The great majority of the zirconia implants used in the studies were of the type of one-piece implant, which has some advantages. One-piece implants were developed to integrate the transmucosal abutment as an integral element of the implant, providing benefits such as the absence of micro gaps between the implant and abutment, decreased microbial accumulation [72], reduced surgical time, and a simple restorative approach [73]. They are intended for immediate loading as well as immediate insertion following tooth extraction and can be surgically implanted with or without a flap [73].

There is, however, other possible reason for the preference of one-piece implants over the two-piece ones when it comes specifically to implants manufactured of zirconia. It can be hypothesized that the implant manufacture industry was initially skeptical that the thin parts associated with the prosthetic connection of a two-piece implant of zirconia would properly tolerate loads in the same way as the long-tested implants of titanium alloys. Therefore, most (if not all) of the zirconia implants initially available in the market were of the one-piece type. In fact, the first clinical study on zirconia implants was published in 2006, while the first one to include two-piece zirconia implants was published in 2014. Until this date, not so many studies evaluating two-piece zirconia implants have been published. Only 4 out of the 25 studies included in the review evaluated two-piece zirconia implants. Fractures of dental implants usually originate either at the abutment neck, the internal connection between the abutment and the implant, or the inner thread of two-piece implants. The fracture origins are associated with damages at the abutment surface of two-piece implants [74]. One-piece implants can, according to in vitro studies, be considered more fracture resistant than two-piece implants [66]. Making the implant in two parts would make the prosthetic connections with very thin structures in some regions, which could allegedly make it more susceptible to fractures [26]. In fact, most zirconia implants are manufactured as one-piece implant systems because of the limitations of the material [19].

The mean MBL remained between 0.632 and 2.060 mm over long periods of observation, namely, up until 132 months, showing similar MBL results of those observed by titanium alloys implants [3, 4, 27, 75]. The fluctuation of the MBL mean values in different time points may be due to differences in sample size in different follow-ups, as well as of different implant configurations [4]. Despite the fluctuation, the mean values did not vary much from an initial moderate MBL during the first months of function, which may represent normal bone remodeling in response to surgery of implants installation, not necessarily a sign of pathology [27].

### Limitations of the present systematic review

The results of the present review have to be interpreted with caution because of its limitations. First of all, all confounding factors may have affected the long-term outcomes. The included studies have a considerable number of confounding factors, and most of the studies, if not all, did not inform how many implants were inserted and survived/lost in several different conditions. For example, studies reported the presence of smokers and bruxers among the patients, as well as diabetic patients, patients with a history of periodontitis, implants placed in fresh extraction sockets, factors that may have a considerable impact on implant failure rates [76–82]. The impact of these variables on the implant survival rate is difficult to estimate if these factors are not identified separately between the different implant groups in order to perform a meta-regression analysis. The real fact is that individual patients sometimes present with more than one risk factor [25, 83], and groups of patients are typically heterogeneous with respect to risk factors and susceptibilities so the specific effect of an individual risk factor could be isolated neither for individual studies nor for the present review.

Second, most of the included studies had a retrospective design, and the nature of a retrospective study inherently results in flaws. These problems were manifested by the gaps in information and incomplete records.

Third, much of the research in the field is limited by small cohort size and short follow-up periods.

## Conclusion

Zirconia implants present high 10-year cumulative survival rate and short-term low marginal bone loss. Despite the increasing number of clinical studies published recently, most of them are of limited (≤ 60 months) follow-up.

### Supplementary Information

Below is the link to the electronic supplementary material.

Supplementary file1 (PDF 923 KB)

## Data Availability

This is a systematic review and all the available data is presented in the article. Moreover, all the included studies were listed, from where the data can also be retrieved.

## References

[1] Depprich R, Zipprich H, Ommerborn M, Naujoks C, Wiesmann HP, Kiattavorncharoen S, Lauer HC, Meyer U, Kübler NR, Handschel J (2008). Osseointegration of zirconia implants compared with titanium: an in vivo study. Head Face Med.

[2] Steinemann SG (2000). (1998) Titanium–the material of choice?. Periodontol.

[3] Chrcanovic BR, Kisch J, Albrektsson T, Wennerberg A (2018). A retrospective study on clinical and radiological outcomes of oral implants in patients followed up for a minimum of 20 years. Clin Implant Dent Relat Res.

[4] Wennerberg A, Albrektsson T, Chrcanovic B (2018). Long-term clinical outcome of implants with different surface modifications. Eur J Oral Implantol.

[5] Ioannidis A, Cathomen E, Jung RE, Fehmer V, Hüsler J, Thoma DS (2017). Discoloration of the mucosa caused by different restorative materials - a spectrophotometric in vitro study. Clin Oral Implant Res.

[6] Thoma DS, Ioannidis A, Cathomen E, Hämmerle CH, Hüsler J, Jung RE (2016). Discoloration of the peri-implant mucosa caused by zirconia and titanium implants. Int J Periodontics Restorative Dent.

[7] Heydecke G, Kohal R, Gläser R (1999). Optimal esthetics in single-tooth replacement with the Re-Implant system: a case report. Int J Prosthodont.

[8] Kohal RJ, Klaus G (2004). A zirconia implant-crown system: a case report. Int J Periodontics Restorative Dent.

[9] Albrektsson T, Chrcanovic B, Molne J, Wennerberg A (2018). Foreign body reactions, marginal bone loss and allergies in relation to titanium implants. Eur J Oral Implantol.

[10] Gawkrodger DJ (2005). Investigation of reactions to dental materials. Br J Dermatol.

[11] Hosoki M, Bando E, Asaoka K, Takeuchi H, Nishigawa K (2009). Assessment of allergic hypersensitivity to dental materials. Bio-Med Mater Eng.

[12] Lalor PA, Revell PA, Gray AB, Wright S, Railton GT, Freeman MA (1991). Sensitivity to titanium. A cause of implant failure?. J Bone Joint Surg British.

[13] Viraben R, Boulinguez S, Alba C (1995). Granulomatous dermatitis after implantation of a titanium-containing pacemaker. Contact Dermatitis.

[14] Andreiotelli M, Kohal RJ (2009). Fracture strength of zirconia implants after artificial aging. Clin Implant Dent Relat Res.

[15] Kohal RJ, Wolkewitz M, Mueller C (2010). Alumina-reinforced zirconia implants: survival rate and fracture strength in a masticatory simulation trial. Clin Oral Implant Res.

[16] Gahlert M, Röhling S, Wieland M, Eichhorn S, Küchenhoff H, Kniha H (2010). A comparison study of the osseointegration of zirconia and titanium dental implants. A biomechanical evaluation in the maxilla of pigs. Clin Implant Dent Relat Res.

[17] Gahlert M, Roehling S, Sprecher CM, Kniha H, Milz S, Bormann K (2012). In vivo performance of zirconia and titanium implants: a histomorphometric study in mini pig maxillae. Clin Oral Implant Res.

[18] Bosshardt DD (2000). Chappuis V and Buser D (2017) Osseointegration of titanium, titanium alloy and zirconia dental implants: current knowledge and open questions. Periodontol.

[19] Cionca N (2000). Hashim D and Mombelli A (2017) Zirconia dental implants: where are we now, and where are we heading?. Periodontol.

[20] Roehling S, Gahlert M, Bacevic M, Woelfler H, Laleman I (2023). Clinical and radiographic outcomes of zirconia dental implants-a systematic review and meta-analysis. Clin Oral Implant Res.

[21] Page MJ, Moher D, Bossuyt PM, Boutron I, Hoffmann TC, Mulrow CD, Shamseer L, Tetzlaff JM, Akl EA, Brennan SE, Chou R, Glanville J, Grimshaw JM, Hróbjartsson A, Lalu MM, Li T, Loder EW, Mayo-Wilson E, McDonald S, McGuinness LA, Stewart LA, Thomas J, Tricco AC, Welch VA, Whiting P, McKenzie JE (2021). PRISMA 2020 explanation and elaboration: updated guidance and exemplars for reporting systematic reviews. BMJ (Clinical Research Ed).

[22] NIH (2014) Quality assessment tool for case series studies. National Institutes of Health (NIH). https://www.nhlbi.nih.gov/health-topics/study-quality-assessment-tools. Accessed 29 Aug 2022

[23] Manicone PF, Rossi Iommetti P, Raffaelli L, Paolantonio M, Rossi G, Berardi D, Perfetti G (2007). Biological considerations on the use of zirconia for dental devices. Int J Immunopathol Pharmacol.

[24] Tonetti MS, Schmid J (1994). Pathogenesis of implant failures. Periodontol.

[25] Chrcanovic BR, Kisch J, Albrektsson T, Wennerberg A (2016). Factors influencing early dental implant failures. J Dent Res.

[26] Chrcanovic BR, Kisch J, Albrektsson T, Wennerberg A (2018). Factors influencing the fracture of dental implants. Clin Implant Dent Relat Res.

[27] Albrektsson T, Chrcanovic B (2000). Östman PO and Sennerby L (2017) Initial and long-term crestal bone responses to modern dental implants. Periodontol.

[28] DerSimonian R, Laird N (1986). Meta-analysis in clinical trials. Control Clin Trials.

[29] Wallace BC, Dahabreh IJ, Trikalinos TA, Lau J, Trow P, Schmid CH (2012). Closing the gap between methodologists and end-users: R as a computational back-end. J Stat Softw.

[30] Balmer M, Spies BC, Kohal RJ, Hammerle CH, Vach K, Jung RE (2020). Zirconia implants restored with single crowns or fixed dental prostheses: 5-year results of a prospective cohort investigation. Clin Oral Implant Res.

[31] Becker J, John G, Becker K, Mainusch S, Diedrichs G, Schwarz F (2017). Clinical performance of two-piece zirconia implants in the posterior mandible and maxilla: a prospective cohort study over 2 years. Clin Oral Implant Res.

[32] Blaschke C, Volz U (2006). Soft and hard tissue response to zirconium dioxide dental implants–a clinical study in man. Neuro Endocrinol Lett.

[33] Borgonovo AE, Censi R, Vavassori V, Arnaboldi O, Maiorana C, Re D (2015). Zirconia implants in esthetic areas: 4-year follow-up evaluation study. Int J Dent.

[34] Brüll F, van Winkelhoff AJ, Cune MS (2014). Zirconia dental implants: a clinical, radiographic, and microbiologic evaluation up to 3 years. Int J Oral Maxillofac Implants.

[35] Cannizzaro G, Torchio C, Felice P, Leone M, Esposito M (2010). Immediate occlusal versus non-occlusal loading of single zirconia implants. A multicentre pragmatic randomised clinical trial. Eur J Oral Implantol.

[36] Cionca N, Hashim D, Mombelli A (2021). Two-piece zirconia implants supporting all-ceramic crowns: six-year results of a prospective cohort study. Clin Oral Implant Res.

[37] Gahlert M, Kniha H, Laval S, Gellrich NC, Bormann KH (2022). Prospective clinical multicenter study evaluating the 5-year performance of zirconia implants in single-tooth gaps. Int J Oral Maxillofac Implants.

[38] Grassi FR, Capogreco M, Consonni D, Bilardi G, Buti J, Kalemaj Z (2015). Immediate occlusal loading of one-piece zirconia implants: five-year radiographic and clinical evaluation. Int J Oral Maxillofac Implants.

[39] Hagi D (2021). Stability determination of one-piece ceramic implants using the periotest device: follow-up study of up to 12 months. Int J Oral Maxillofac Implants.

[40] Kiechle S, Liebermann A, Mast G, Heitzer M, Möhlhenrich SC, Hölzle F, Kniha H, Kniha K (2023). Evaluation of one-piece zirconia dental implants: an 8-year follow-up study. Clin Oral Invest.

[41] Kniha K, Milz S, Kniha H, Ayoub N, Hölzle F, Modabber A (2018). Peri-implant crestal bone changes around zirconia implants in periodontally healthy and compromised patients. Int J Oral Maxillofac Implants.

[42] Kohal RJ, Burkhardt F, Chevalier J, Patzelt SBM, Butz F (2023). One-piece zirconia oral implants for single tooth replacement: five-year results from a prospective cohort study. J Func Biomater.

[43] Kohal RJ, Spies BC, Vach K, Balmer M, Pieralli S (2020). A Prospective clinical cohort investigation on zirconia implants: 5-year results. J Clin Med.

[44] Kohal RJ, Vach K, Butz F, Spies BC, Patzelt SBM, Burkhardt F (2023). One-piece zirconia oral implants for the support of three-unit fixed dental prostheses: three-year results from a prospective case series. J Func Biomater.

[45] Koller M, Steyer E, Theisen K, Stagnell S, Jakse N, Payer M (2020). Two-piece zirconia versus titanium implants after 80 months: clinical outcomes from a prospective randomized pilot trial. Clin Oral Implant Res.

[46] Kunavisarut C, Buranajanyakul L, Kitisubkanchana J, Pumpaluk P (2020). A pilot study of small-diameter one-piece ceramic implants placed in anterior regions: clinical and esthetic outcomes at 1-year follow-up. Int J Oral Maxillofac Implants.

[47] Lorenz J, Giulini N, Hölscher W, Schwiertz A, Schwarz F, Sader R (2019). Prospective controlled clinical study investigating long-term clinical parameters, patient satisfaction, and microbial contamination of zirconia implants. Clin Implant Dent Relat Res.

[48] Oliva J, Oliva X (2023). 15-year post-market clinical follow-up study of 1,828 ceramic (zirconia) implants in humans. Int J Oral Maxillofac Implants.

[49] Oliva J, Oliva X, Oliva JD (2010). Five-year success rate of 831 consecutively placed Zirconia dental implants in humans: a comparison of three different rough surfaces. Int J Oral Maxillofac Implants.

[50] Osman RB, Swain MV, Atieh M, Ma S, Duncan W (2014). Ceramic implants (Y-TZP): are they a viable alternative to titanium implants for the support of overdentures? A randomized clinical trial. Clin Oral Implant Res.

[51] Roehling S, Woelfler H, Hicklin S, Kniha H, Gahlert M (2016). A retrospective clinical study with regard to survival and success rates of zirconia implants up to and after 7 years of loading. Clin Implant Dent Relat Res.

[52] Ruiz Henao PA, Caneiro Queija L, Mareque S, Tasende Pereira A, Linares Gonzalez A, Blanco Carrion J (2021). Titanium vs ceramic single dental implants in the anterior maxilla: a 12-month randomized clinical trial. Clin Oral Implant Res.

[53] Steyer E, Herber V, Koller M, Vegh D, Mukaddam K, Jakse N, Payer M (2021) Immediate restoration of single-piece zirconia implants: a prospective case series-long-term results after 11 years of clinical function. Materials (Basel, Switzerland) 14. 10.3390/ma14226738 10.3390/ma14226738 PMC8621133 34832139

[54] Vilor-Fernandez M, Garcia-De-La-Fuente AM, Marichalar-Mendia X, Estefania-Fresco R, Aguirre-Zorzano LA (2021). Single tooth restoration in the maxillary esthetic zone using a one-piece ceramic implant with 1 year of follow-up: case series. Int J Implant Dent.

[55] Layton DM (2013). Understanding Kaplan-Meier and survival statistics. Int J Prosthodont.

[56] Ferguson JG (1992). Life tables for clinical scientists. J Vasc Interv Radiol.

[57] Kohal RJ, Wolkewitz M, Tsakona A (2011). The effects of cyclic loading and preparation on the fracture strength of zirconium-dioxide implants: an in vitro investigation. Clin Oral Implant Res.

[58] Kiliaridis S, Kjellberg H, Wenneberg B, Engström C (1993). The relationship between maximal bite force, bite force endurance, and facial morphology during growth A cross-sectional study. Acta Odontol Scand.

[59] Ferrario VF, Sforza C, Zanotti G, Tartaglia GM (2004). Maximal bite forces in healthy young adults as predicted by surface electromyography. J Dent.

[60] Fontijn-Tekamp FA, Slagter AP, Van Der Bilt A, Van THMA, Witter DJ, Kalk W, Jansen JA (2000). Biting and chewing in overdentures, full dentures, and natural dentitions. J Dent Res.

[61] Osborn JW (1990). Anterior component of force. Am J Orthod Dentofac Orthop.

[62] Nishigawa K, Bando E, Nakano M (2001). Quantitative study of bite force during sleep associated bruxism. J Oral Rehabil.

[63] Tinschert J, Natt G, Körbe S, Neines N, Heussen N, Weber M, Spiekermann H (2006). Bruchfestigkeit zirkonoxidbasierter Seitenzahnbrücken. Eine vergleichende In-vitro-Studie Quintessenz Zahnmedizin.

[64] Cales B, Stefani Y, Lilley E (1994). Long-term in vivo and in vitro aging of a zirconia ceramic used in orthopaedy. J Biomed Mater Res.

[65] Gahlert M, Burtscher D, Grunert I, Kniha H, Steinhauser E (2012). Failure analysis of fractured dental zirconia implants. Clin Oral Implant Res.

[66] Bethke A, Pieralli S, Kohal RJ, Burkhardt F, von Stein-Lausnitz M, Vach K, Spies BC (2020). Fracture resistance of zirconia oral implants in vitro: a systematic review and meta-analysis. Materials (Basel, Switzerland).

[67] Atalay P, Öztaş DD (2022). Fatigue resistance and fracture strength of narrow-diameter one-piece zirconia implants with angled abutments. J Esthet Restor Dent.

[68] Scherrer SS, Mekki M, Crottaz C, Gahlert M, Romelli E, Marger L, Durual S, Vittecoq E (2019). Translational research on clinically failed zirconia implants. Dental Mater.

[69] Guazzato M, Quach L, Albakry M, Swain MV (2005). Influence of surface and heat treatments on the flexural strength of Y-TZP dental ceramic. J Dent.

[70] Kosmac T, Oblak C, Jevnikar P, Funduk N, Marion L (1999). The effect of surface grinding and sandblasting on flexural strength and reliability of Y-TZP zirconia ceramic. Dental Mater.

[71] Denry IL, Holloway JA (2006). Microstructural and crystallographic surface changes after grinding zirconia-based dental ceramics. J Biomed Mater Res B Appl Biomater.

[72] Broggini N, McManus LM, Hermann JS, Medina RU, Oates TW, Schenk RK, Buser D, Mellonig JT, Cochran DL (2003). Persistent acute inflammation at the implant-abutment interface. J Dent Res.

[73] Prithviraj DR, Gupta V, Muley N, Sandhu P (2013). One-piece implants: placement timing, surgical technique, loading protocol, and marginal bone loss. J Prosthodont.

[74] Zhang F, Monzavi M, Li M, Čokić S, Manesh A, Nowzari H, Vleugels J, Van Meerbeek B (2022). Fracture analysis of one/two-piece clinically failed zirconia dental implants. Dental Mater.

[75] Chrcanovic BR, Albrektsson T, Wennerberg A (2016). Turned versus anodised dental implants: a meta-analysis. J Oral Rehabil.

[76] Al Ansari Y, Shahwan H, Chrcanovic BR (2022). Diabetes mellitus and dental implants: a systematic review and meta-analysis. Materials (Basel, Switzerland).

[77] Chrcanovic BR, Albrektsson T, Wennerberg A (2014). Periodontally compromised vs. periodontally healthy patients and dental implants: a systematic review and meta-analysis. J Dent.

[78] Chrcanovic BR, Kisch J, Albrektsson T, Wennerberg A (2016). Bruxism and dental implant failures: a multilevel mixed effects parametric survival analysis approach. J Oral Rehabil.

[79] Chrcanovic BR, Kisch J, Albrektsson T, Wennerberg A (2017). Bruxism and dental implant treatment complications: a retrospective comparative study of 98 bruxer patients and a matched group. Clin Oral Implant Res.

[80] Ibrahim A, Chrcanovic BR (2021). Dental implants inserted in fresh extraction sockets versus healed sites: a systematic review and meta-analysis. Materials.

[81] Mustapha AD, Salame Z, Chrcanovic BR (2021). Smoking and dental implants: a systematic review and meta-analysis. Medicina (Kaunas).

[82] Haggman-Henrikson B, Ali D, Aljamal M, Chrcanovic BR (2023). Bruxism and dental implants: a systematic review and meta-analysis. J Oral Rehabil.

[83] Chrcanovic BR, Kisch J, Albrektsson T, Wennerberg A (2017). Analysis of risk factors for cluster behavior of dental implant failures. Clin Implant Dent Relat Res.

