# Supplementary material for: Clinical outcomes of zirconia implants: a systematic review and meta-analysis
Source: Clin Oral Investig. 2023 Dec 23;28(1):15. doi: 10.1007/s00784-023-05401-8 (PMC10746607; doi:10.1007/s00784-023-05401-8)
Supplement: Supplementary file 1 — Supplementary file1 (PDF 923 KB) [file 784_2023_5401_MOESM1_ESM.pdf]

SUPPLEMENTARY MATERIAL

- **a. Reference list of included publications**
- **b. Table S1. Detailed data of the included studies**
- **c. Table S2. Quality assessment**
- **d. Forest plots for the outcome marginal bone loss**

a. <u>Reference list of included publications</u>

1. Balmer M, Spies BC, Kohal RJ, Hämmerle CH, Vach K, Jung RE. Zirconia implants restored with single crowns or fixed dental prostheses: 5-year results of a prospective cohort investigation. Clin Oral Implants Res. 2020 May;31(5):452-462.
2. Becker J, John G, Becker K, Mainusch S, Diedrichs G, Schwarz F. Clinical performance of two-piece zirconia implants in the posterior mandible and maxilla: a prospective cohort study over 2 years. Clin Oral Implants Res. 2017 Jan;28(1):29-35.
3. Blaschke C, Volz U. Soft and hard tissue response to zirconium dioxide dental implants--a clinical study in man. Neuro Endocrinol Lett. 2006 Dec;27 Suppl 1:69-72.
4. Borgonovo AE, Censi R, Vavassori V, Arnaboldi O, Maiorana C, Re D. Zirconia Implants in Esthetic Areas: 4-Year Follow-Up Evaluation Study. Int J Dent. 2015;2015:415029.
5. Brüll F, van Winkelhoff AJ, Cune MS. Zirconia dental implants: a clinical, radiographic, and microbiologic evaluation up to 3 years. Int J Oral Maxillofac Implants. 2014 Jul-Aug;29(4):914-20.
6. Cannizzaro G, Torchio C, Felice P, Leone M, Esposito M. Immediate occlusal versus non-occlusal loading of single zirconia implants. A multicentre pragmatic randomised clinical trial. Eur J Oral Implantol. 2010 Summer;3(2):111-20.
7. Cionca N, Hashim D, Mombelli A. Two-piece zirconia implants supporting all-ceramic crowns: Six-year results of a prospective cohort study. Clin Oral Implants Res. 2021 Jun;32(6):695-701.
8. Gahlert M, Kniha H, Laval S, Gellrich NC, Bormann KH. Prospective Clinical Multicenter Study Evaluating the 5-Year Performance of Zirconia Implants in Single-Tooth Gaps. Int J Oral Maxillofac Implants. 2022 Jul-Aug;37(4):804-811.
9. Grassi FR, Capogreco M, Consonni D, Bilardi G, Buti J, Kalemaj Z. Immediate occlusal loading of one-piece zirconia implants: five-year radiographic and clinical evaluation. Int J Oral Maxillofac Implants. 2015 May-Jun;30(3):671-80.
10. Hagi D. Stability Determination of One-Piece Ceramic Implants Using the Periotest Device: Follow-up Study of Up to 12 Months. Int J Oral Maxillofac Implants. 2021 Jul-Aug;36(4):738-744.
11. Kiechle S, Liebermann A, Mast G, Heitzer M, Möhlhenrich SC, Hölzle F, Kniha H, Kniha K. Evaluation of one-piece zirconia dental implants: An 8-year follow-up study. Clin Oral Investig. 2023 Jun 5.
12. Kniha K, Milz S, Kniha H, Ayoub N, Hölzle F, Modabber A. Peri-implant Crestal Bone Changes Around Zirconia Implants in Periodontally Healthy and Compromised Patients. Int J Oral Maxillofac Implants. 2018 January/February;33(1):217–222.
13. Kohal RJ, Burkhardt F, Chevalier J, Patzelt SBM, Butz F. One-Piece Zirconia Oral Implants for Single Tooth Replacement: Five-Year Results from a Prospective Cohort Study. J Funct Biomater. 2023 Feb 19;14(2):116.
14. Kohal RJ, Spies BC, Vach K, Balmer M, Pieralli S. A Prospective Clinical Cohort Investigation on Zirconia Implants: 5-Year Results. J Clin Med. 2020 Aug 10;9(8):2585.
15. Kohal RJ, Vach K, Butz F, Spies BC, Patzelt SBM, Burkhardt F. One-Piece Zirconia Oral Implants for the Support of Three-Unit Fixed Dental Prostheses: Three-Year Results from a Prospective Case Series. J Funct Biomater. 2023 Jan 13;14(1):45.

16. Koller M, Steyer E, Theisen K, Stagnell S, Jakse N, Payer M. Two-piece zirconia versus titanium implants after 80 months: Clinical outcomes from a prospective randomized pilot trial. Clin Oral Implants Res. 2020 Apr;31(4):388-396.
17. Kunavisarut C, Buranajanyakul L, Kitisubkanchana J, Pumpaluk P. A Pilot Study of Small-Diameter One-Piece Ceramic Implants Placed in Anterior Regions: Clinical and Esthetic Outcomes at 1-Year Follow-up. Int J Oral Maxillofac Implants. 2020 Sep/Oct;35(5):965-973.
18. Lorenz J, Giulini N, Hölscher W, Schwiertz A, Schwarz F, Sader R. Prospective controlled clinical study investigating long-term clinical parameters, patient satisfaction, and microbial contamination of zirconia implants. Clin Implant Dent Relat Res. 2019 Apr;21(2):263-271.
19. Oliva J, Oliva X, Oliva JD. Five-year success rate of 831 consecutively placed Zirconia dental implants in humans: a comparison of three different rough surfaces. Int J Oral Maxillofac Implants. 2010 Mar-Apr;25(2):336-44.
20. Oliva J, Oliva X. 15-Year Post-Market Clinical Follow-up Study of 1,828 Ceramic (Zirconia) Implants in Humans. Int J Oral Maxillofac Implants. 2023 Mar-Apr;38(2):357-366.
21. Osman RB, Swain MV, Atieh M, Ma S, Duncan W. Ceramic implants (Y-TZP): are they a viable alternative to titanium implants for the support of overdentures? A randomized clinical trial. Clin Oral Implants Res. 2014 Dec;25(12):1366-77.
22. Roehling S, Woelfler H, Hicklin S, Kniha H, Gahlert M. A Retrospective Clinical Study with Regard to Survival and Success Rates of Zirconia Implants up to and after 7 Years of Loading. Clin Implant Dent Relat Res. 2016 Jun;18(3):545-58.
23. Ruiz Henao PA, Caneiro Queija L, Mareque S, Tasende Pereira A, Liñares González A, Blanco Carrión J. Titanium vs ceramic single dental implants in the anterior maxilla: A 12-month randomized clinical trial. Clin Oral Implants Res. 2021 Aug;32(8):951-961.
24. Steyer E, Herber V, Koller M, Végh D, Mukaddam K, Jakse N, Payer M. Immediate Restoration of Single-Piece Zirconia Implants: A Prospective Case Series-Long-Term Results after 11 Years of Clinical Function. Materials (Basel). 2021 Nov 9;14(22):6738.
25. Vilor-Fernández M, García-De-La-Fuente AM, Marichalar-Mendia X, Estefanía-Fresco R, Aguirre-Zorzano LA. Single tooth restoration in the maxillary esthetic zone using a one-piece ceramic implant with 1 year of follow-up: case series. Int J Implant Dent. 2021 Apr 6;7(1):26. doi: 10.1186/s40729-021-00308-z. Erratum in: Int J Implant Dent. 2021 Nov 24;7(1):114.

b. Detailed data of the included studies

Table S1. Detailed data of the included studies.

Study	Year	Study design	Country / Setting	Patients (male/ female) (n)	Patients' Age Range (mean) (years)	Implant healing period	Implants location	Implant used	Prosthesis type	Follow-up (months)
Balmer	2020	PS/MU	Germany, Switzerland / University	60 (30/30)	NA (48.1)	4 mo (Mx) 2 mo (Md)	Mx, Md Ant/Post	ceramic.implant (VITA Zahnfabrik, Bad Säckingen, Germany)	SC, partial FDP	60
Becker	2017	PS/UN	Germany / University	48 (17/31)	NA (47.6)	12 wk (Mx) 10 wk (Md)	Mx, Md Post	ZV3 (Zircon Vision GmbH, Wolfrathausen, Germany)	SC	32.9 (mean)
Blaschke	2006	RS/UN	Germany / University	34 (NA)	NA	6 mo (Mx) 4 mo (Md)	Mx, Md Ant/Post	VOLZIRKON1, VOLZIRKON2, Z-Lock3 (Z-Systems AG, Constance, Germany)	NA	12-24
Borgonovo	2015	RS/UN	Italy / University	14 (13/1)	38-75 (60)	Immediate loading	Mx Ant	White-SKY (Bredent Senden, Germany)	SC, partial FDP	48
Brüll	2014	RS/UN	Netherlands / University	74 (17/57)	18-72 (51)	3-17 mo	Mx, Md Ant/Post	Own manufactured implants	SC, partial FDP	18 (mean)
Cannizzaro	2010	PS/MU	Italy / Private practice	40 (17/23)	18-55 (38)	Immediate loading	Mx, Md Ant/Post	Z-Look 3 (Z-Systems, Oensingen, Switzerland)	SC	12
Cionca	2021	PS/UN	Switzerland / University	24 (11/13)	24-75 (51.9)	3 mo	Mx, Md Post	Zeramex T (Dentalpoint AG, Spreitenbach, Switzerland)	SC	82.2 (mean)
Gahlert	2022	PS/MU	Germany / Private practice, Hospital	36 (15/21)	24-83 (53)	11-13 wk	Mx, Md Ant/Post	PURE Ceramic (Straumann, Basel, Switzerland)	SC	60
Grassi	2015	PS/MU	Italy / University, Private practice	17 (8/9)	35-70 (52.3)	Immediate loading	Mx, Md Ant/Post	White-SKY (Bredent Senden, Germany)	SC	60
Hagi	2021	RS/UN	Canada / Private practice	202 (77/125)	19-79 (NA)	Immediate Delayed	Mx, Md Ant/Post	PURE Ceramic (Straumann, Basel, Switzerland) CeraRoot ICE (CeraRoot, Santa Monica, USA)	SC, partial FDP	≤ 12
Kiechle	2023	PS/UN	Germany / Private practice	39 (NA)	29-84 (58.8)	Immediate Delayed	Mx Ant	PURE Ceramic (Straumann, Basel, Switzerland)	SC	96
Kniha	2018	PS/UN	Germany / University	86 (35/49)	25-67 (55)	3-5 mo	Mx, Md Ant/Post	PURE Ceramic (Straumann, Basel, Switzerland)	SC	15-18

Kohal	2020	PS/UN	Germany / University	35 (NA)	18-70 (NA)	Immediate	Mx, Md Ant/Post	Ziraldent FR1 (Metoxit AG, Thayngen, Switzerland)	SC, partial FDP	60
Kohal	2023a	PS/UN	Germany / University	65 (NA)	18-70 (NA)	Immediate	Mx, Md Ant/Post	ZiUnite (Nobel Biocare AB, Göteborg, Sweden)	SC	60
Kohal	2023b	PS/UN	Germany / University	27 (11/16)	18-70 (NA)	Immediate	Mx, Md Ant/Post	ZiUnite (Nobel Biocare AB, Göteborg, Sweden)	partial FDP	36
Koller	2020	PS/UN	Austria / University	22 (13/9)	24-77 (46)	6 mo (Mx) 4 mo (Md)	Mx, Md Ant/Post	Ziterion Vario Z (Ziterion Gmbh, Uffenheim, Germany)	SC	80
Kunavisarut	2020	PS/UN	Thailand / University	20 (6/14)	25-72 (52.6)	Immediate	Mx, Md Ant	PURE Ceramic (Straumann, Basel, Switzerland)	SC	12
Lorenz	2019	PS/MU	Germany / University, Private practice	28 (13/15)	39-80 (63.5)	6 mo (Mx) 4 mo (Md)	Mx, Md Ant/Post	Z-Look 3 (Z-Systems, Oensingen, Switzerland)	SC	93.6 (mean)
Oliva [a]	2010	RS/UN	Spain / Private practice	378 (151/227)	19-80 (48.18)	Immediate Delayed	Mx, Md Ant/Post	CeraRoot UC and C (CeraRoot, Santa Monica, USA)	SC, partial FDP	40.8 (mean)
Oliva	2023	RS/UN	Spain / Private practice	771 (308/463)	19-90 (51.18)	Immediate Delayed	Mx, Md Ant/Post	CeraRoot ICE (CeraRoot, Santa Monica, USA)	SC, partial FDP	106.4 (mean)
Osman	2014	PS/UN	New Zealand / University	12 (NA)	46-80 (62)	4 mo	Mx, Md Ant/Post	(Southern Implants, Irene, South Africa)	Overdenture	12
Roehling	2016	RS/UN	Germany / Private practice	71 (32/39)	19-85 (54.86)	Delayed	Mx, Md Ant/Post	Z-Look 3 (Z-Systems, Kiel, Germany)	SC, partial FDP	71.3 (mean)
Ruiz Henao	2021	PS/UN	Spain / University	16 (4/12)	NA (54.13)	4 days	Mx Ant	PURE Ceramic (Straumann, Basel, Switzerland)	SC	12
Steyer	2021	PS/UN	Austria / University	20 (12/8)	26-70 (43.3)	Immediate	Mx, Md Ant/Post	White-SKY (Bredent Senden, Germany)	SC	96-132
Vilor-Fernández	2021	PS/UN	Spain / University	28 (12/16)	34-67 ()	Immediate Delayed (6-8 wk)	Mx Ant	PURE Ceramic (Straumann, Basel, Switzerland)	SC	12

NA – not available; RS – retrospective study; PS – prospective study; UN – unicenter; MU – multicenter
wk – weeks; mo – months
Mx – maxilla; Md – mandible; Ant – anterior region; Post – posterior region
SC – single crown; FDP – fixed dental prosthesis
[a] CeraRoot ICE implants from this study were not included, due to possible overlap with the same implant type in Oliva et al. (2023)

c. Quality assessment

Table S2. Quality assessment tool, according to the National Institutes of Health (NIH)

Study	Year	Was the study question or objective clearly stated?	Was the study population clearly and fully described, including a case definition?	Were the cases consecutive?	Were the subjects comparable?	Was the intervention clearly described?	Were the outcome measures clearly defined, valid, reliable, and implemented consistently across all study participants?	Was the length of follow-up adequate? [a]	Were the statistical methods well-described?	Were the results well-described?	Total (n/9)
Balmer	2020	1	1	0	1	1	1	1	1	1	8/9
Becker	2017	1	1	0	1	1	1	1	1	1	8/9
Blaschke	2006	1	1	1	1	1	1	1	0	0	7/9
Borgonovo	2015	1	1	1	1	1	1	1	0	1	8/9
Brüll	2014	1	1	1	1	1	1	1	1	1	9/9
Cannizzaro	2010	1	1	1	1	1	1	1	1	1	9/9
Cionca	2021	1	1	1	1	1	1	1	1	1	9/9
Gahlert	2022	1	1	1	1	1	1	1	1	1	9/9
Grassi	2015	1	1	0	1	1	1	1	1	1	8/9
Hagi	2021	1	1	1	1	1	1	1	0	1	8/9
Kiechle	2023	1	1	1	1	1	1	1	1	1	9/9
Kniha	2018	1	1	1	1	1	1	1	0	1	8/9
Kohal	2020	1	1	1	1	1	1	1	1	1	9/9
Kohal	2023a	1	1	1	1	1	1	1	1	1	9/9
Kohal	2023b	1	1	1	1	1	1	1	1	1	9/9
Koller	2020	1	1	1	1	1	1	1	1	1	9/9
Kunavisarut	2020	1	1	1	1	1	1	1	1	1	9/9
Lorenz	2019	1	1	0	1	1	1	1	0	1	8/9
Oliva	2010	1	1	1	1	1	1	1	0	0	7/9
Oliva	2023	1	1	1	1	1	1	1	0	0	7/9
Osman	2014	1	1	1	1	1	1	1	1	1	9/9
Roehling	2016	1	1	0	1	1	1	1	0	1	7/9
Ruiz Henao	2021	1	1	1	1	1	1	1	1	1	9/9
Steyer	2021	1	1	0	1	1	1	1	0	1	7/9
Vilor-Fernández	2021	1	1	0	1	1	1	1	0	1	7/9

[a] 3 months of follow-up was chosen to be of adequate length.

d. Forest plots for the outcome marginal bone loss

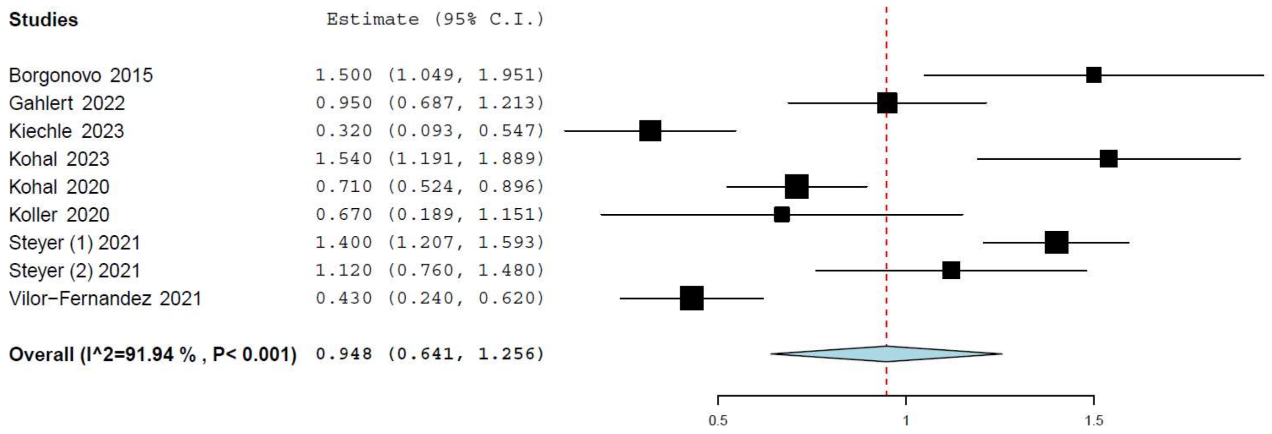

Figure S1. Forest plot for marginal bone loss, 2-6 months of follow-up.

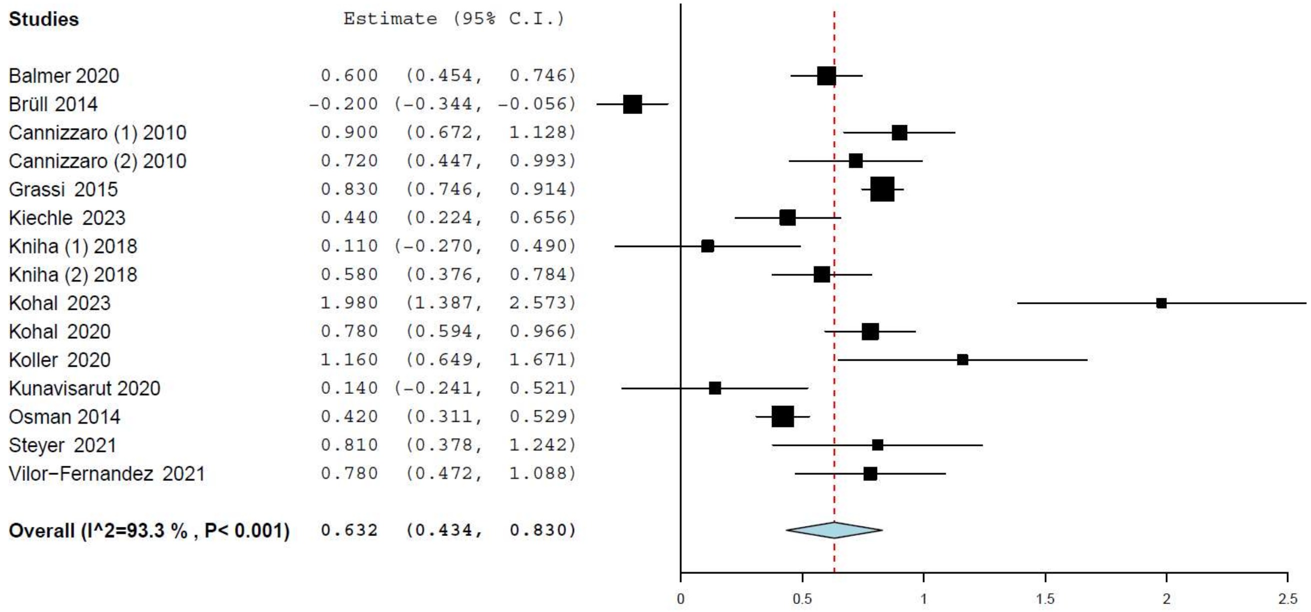

Figure S2. Forest plot for marginal bone loss, 12-15 months of follow-up.

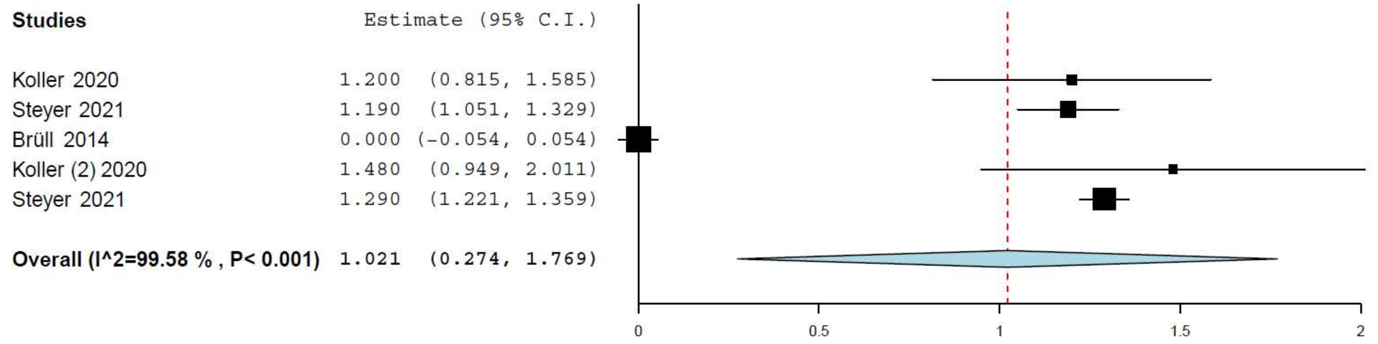

Figure S3. Forest plot for marginal bone loss, 18-24 months of follow-up.

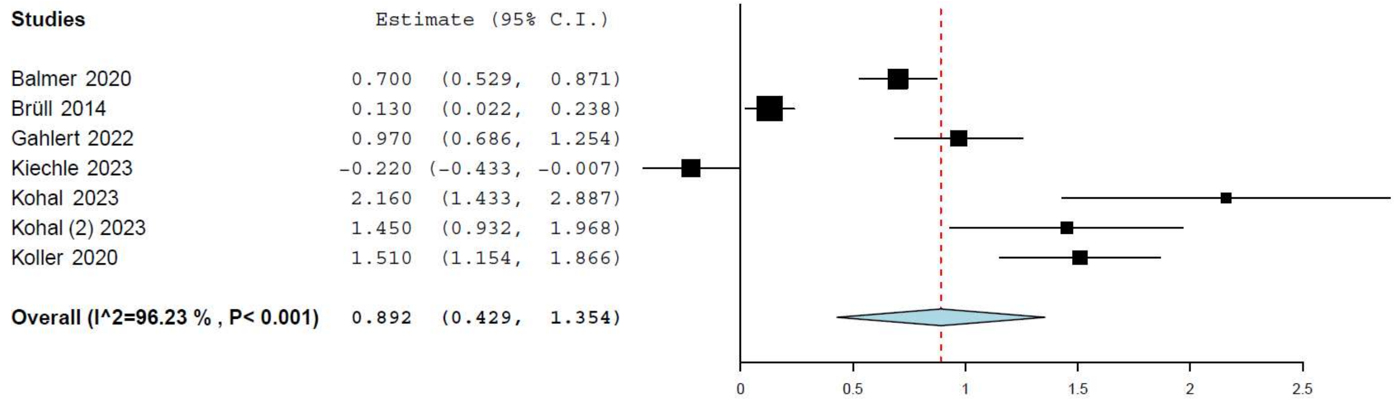

Figure S4. Forest plot for marginal bone loss, 30-36 months of follow-up.

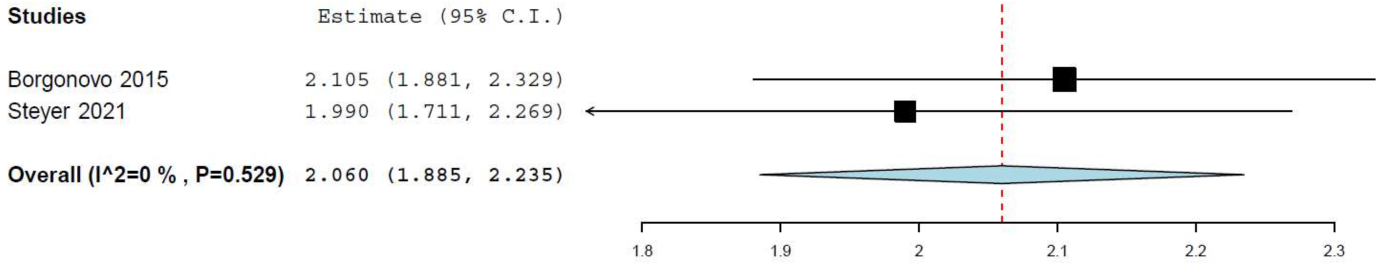

Figure S5. Forest plot for marginal bone loss, 48 months of follow-up.

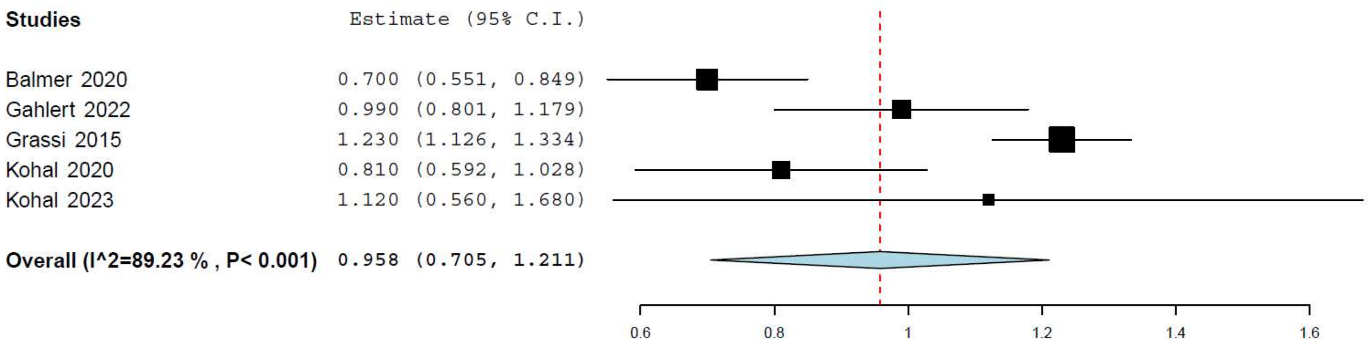

Figure S6. Forest plot for marginal bone loss, 60 months of follow-up.

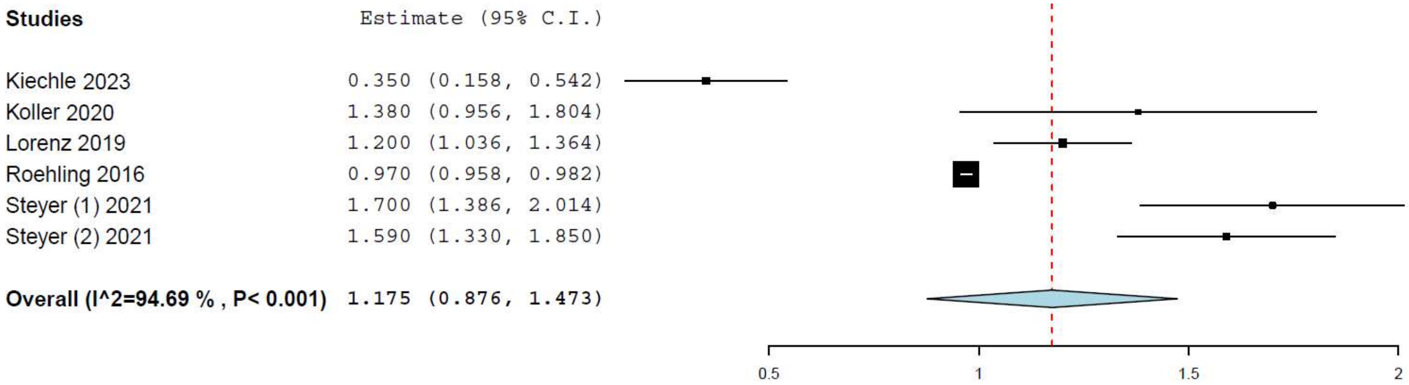

Figure S7. Forest plot for marginal bone loss, 70-132 months of follow-up.
